# Sarcopenia in Ageing and Chronic Illness: Trial Endpoints and Regulatory Issues

**DOI:** 10.1002/jcsm.13841

**Published:** 2025-06-09

**Authors:** Stephan von Haehling, Henning T. Langer, Steven B. Heymsfield, William J. Evans, Stefan D. Anker

**Affiliations:** ^1^ Department of Cardiology and Pneumology University of Göttingen Medical Center Göttingen Germany; ^2^ German Center for Cardiovascular Research (DZHK), Partner Site Göttingen Göttingen Germany; ^3^ Muscle Wasting Laboratory, Department for Geriatrics and Medical Gerontology Charité Universitätsmedizin Berlin, Campus Benjamin Franklin Berlin Germany; ^4^ Metabolism and Body Composition Pennington Biomedical Research Center Baton Rouge Louisiana USA; ^5^ Department of Nutritional Sciences and Toxicology University of California Berkeley Berkeley California USA; ^6^ Division of Geriatrics Duke University Medical Center Durham North Carolina USA; ^7^ Berlin Institute of Health‐Center for Regenerative Therapies (BCRT) Charité‐ Universitätsmedizin Berlin Berlin Germany; ^8^ Deutsches Herzzentrum der Charité, Department of Cardiology (Campus Virchow) Charité Universitätsmedizin Berlin Berlin Germany; ^9^ German Centre for Cardiovascular Research (DZHK) partner site Berlin Berlin Germany

**Keywords:** clinical trials, diagnostics, endpoints, muscle wasting, patient‐reported outcomes, sarcopenia, treatment

## Abstract

In December 2024, the Society on Cachexia and Wasting Disorders (SCWD) hosted a Regulatory and Trial Update Workshop in Washington, D.C., bringing together experts from academia, industry, and the US Food and Drug Administration (FDA). This article summarizes key topics discussed during the meeting, including diagnostic challenges, emerging assessment methods, and trial endpoints. The D_3_‐creatine dilution technique was highlighted as a promising tool for evaluating muscle mass. Additionally, the workshop addressed variability in computed tomography‐based lumbar skeletal muscle index measurements, emphasizing sources of variation at the instrument, imaging, and reader levels, as well as biological and clinical fluctuations. Discussions also focused on clinical trial endpoints for sarcopenia, particularly validated physical performance measures such as the Short Physical Performance Battery (SPPB), habitual gait speed, stair‐climb tests, and the 6‐min walk test. Furthermore, novel therapeutic approaches were explored, including 20‐hydroxyecdysone, enobosarm, anamorelin, ponsegromab, and nutritional supplementation, alongside broader strategies targeting myostatin‐activin signalling inhibition and Akt pathway activation. During the meeting, it was made clear that from a regulatory treatment development standpoint, clinically meaningful changes in patient‐reported outcomes, physical function and/or morbidity/mortality need to be shown. If the latter is not an efficacy endpoint, safety needs to be documented. Given that the population that may be addressed in aging associated sarcopenia is vast, the safety requirement standards applied for studies may be equivalent to those of studies in type 2 diabetes mellitus. Some argued at the meeting that this would make study programs so large that from an economic standpoint only therapies that significantly impact on morbidity/mortality outcomes have a chance to be considered commercially feasible for development.

## 1 Introduction

In December 2024, a “Regulatory and Trial Update Workshop”, organized by the Society on Cachexia and Wasting Disorders (SCWD), was held in Washington D.C. with experts from academia, industry, and the US Food and Drug Administration (FDA) attending. This article summarizes the central messages of December 6, 2024, the second day of the workshop, highlighting key discussion points on trial endpoints in sarcopenia research and regulatory issues in the approval of sarcopenia treatments.

### 1.1 Background

The history of deciphering muscle wasting is a long one. In 1931, Macdonald Critchley, a junior neurologist at King's College Hospital in London, wrote that “the entire musculature tends with advancing age to undergo involutional changes, which are manifested as wasting” [1]. In the 1970s, Nathan Shock published a series of articles on age‐related physiologic functions describing the loss of muscle with ageing. Irwin Rosenberg noted that this phenomenon should be taken seriously, and—at a meeting in Albuquerque, New Mexico, suggested to use the term sarcopenia in 1988 [2]. The term took hold over these last 30 years [3, 4]. Following along these avenues of thought, Prof. William “Bill” Evans (University of California at Berkeley, California, United States) started the first presentation at the Workshop by elaborating that no consensus, no diagnostic criteria and no approved indication exist and—citing his own editorial—asked the question “How did we get here?” [5]. Indeed, despite over 23 000 scientific publications and more than 9000 papers with “sarcopenia” in the title, key challenges remain unresolved, particularly in the areas of diagnostic criteria and functional assessments.

The definition of sarcopenia has varied considerably over time. The first sarcopenia meeting took place in September 1994, sponsored by the National Institute on Aging, and Bill Evans said that the “original definition of sarcopenia was actually pretty simple”, suggesting that age‐related loss of skeletal muscle mass would be predictive of outcomes such as disability and in hip fracture. The problem then started with the first operational definition suggested by Baumgartner in 1998, that identified

$$\text{Sarcopenia}=\frac{\mathrm{ASM}\left(\mathrm{kg}\right)}{\text{Height}{\left(m\right)}^{2}}$$

where ASM is appendicular skeletal muscle mass assessed by dual‐energy X‐ray absorptiometry (DEXA). Sarcopenia was to be diagnosed according to this definition when the height‐adjusted ASM was < 7.26 kg/m^2^ in men or < 5.45 kg/m^2^ in women. There was so much disagreement that the National Institute on Aging put together a foundation who looked at the literature and said, “low lean mass, by itself, is a poor predictor of functional impairment compared with low strength” and “Weakness increases the likelihood of mobility impairment regardless of low lean mass”. In line with this, data in animals [6] and longitudinal studies in humans have later shown that strength is lost at a faster rate than muscle during aging, which has led to the distinction between “dynapenia” (loss in muscle strength) and “sarcopenia” (loss in muscle mass), making the topic rather more difficult [7]. Therefore, Evans said, many groups have moved away from even measuring lean mass because it appears to be unrelated to outcomes.

Later criteria identified sarcopenia through its clinical manifestations, such as increased risk of disability, fractures, and other adverse health outcomes, and have less and less employed muscle mass criteria. Since skeletal muscles are critical for maintaining essential physiological processes, including metabolic regulation, bone integrity, and insulin sensitivity, more recent research indicates that sarcopenia extends beyond muscle mass loss, encompassing factors such as intramyocellular lipids, fibrosis, and neuromuscular dysfunction. These multifaceted interactions complicate efforts to arrive at a universally accepted definition and diagnosis and partly explain why more than 15 definitions of sarcopenia have been published, but not a single one of them uses muscle mass any more for the diagnosis. Bill Evans also highlighted the fact that regulatory bodies seem to be unwilling to consider sarcopenia as an indication for drug therapy, highlighting the need for an outcome trial as well as the need to develop a strategy to choose a more specific indication of older patients with low muscle mass. Participants of the workshop emphasized that while considerable progress has been made in understanding the condition, there remains no consensus on standardized diagnostic criteria. Current approaches rely heavily on muscle mass and functional performance measures, yet discrepancies across methods hinder consistent classification.

### 1.2 Diagnostic Challenges

The workshop delved deeply into the diagnostic obstacles hindering effective sarcopenia management. Early definitions, such as Baumgartner's operational framework linking muscle mass via DEXA to clinical outcomes, are now considered unreliable. Instead, measures of muscle strength (e.g., grip strength) and functional performance (e.g., gait speed) have emerged as stronger correlates of clinical outcomes, including disability and mortality. The difficulties in defining and measuring sarcopenia were highlighted by a meta‐analysis of observational studies, which found prevalence values ranging from 5 to 17%. According to the tool used to assess muscle mass, strength, and physical performance, the prevalence values also varied within definitions from 0 to 22%. Diverse methodologies across studies have led to inconsistent findings, making it difficult to generalize results, leading the authors of the meta‐analysis to conclude that “the establishment of a unique definition for sarcopenia, the use of methods that guarantee an accurate evaluation of muscle mass and the standardization of measurement tools are necessary to allow a proper diagnosis and comparison of sarcopenia prevalence” [8]. One promising approach discussed involves simplified diagnostic criteria that calculate muscle mass relative to body weight. Preliminary studies indicate that this method correlates strongly with adverse outcomes such as mortality and disability, suggesting that it could serve as a practical diagnostic tool.

**FIGURE 1:**
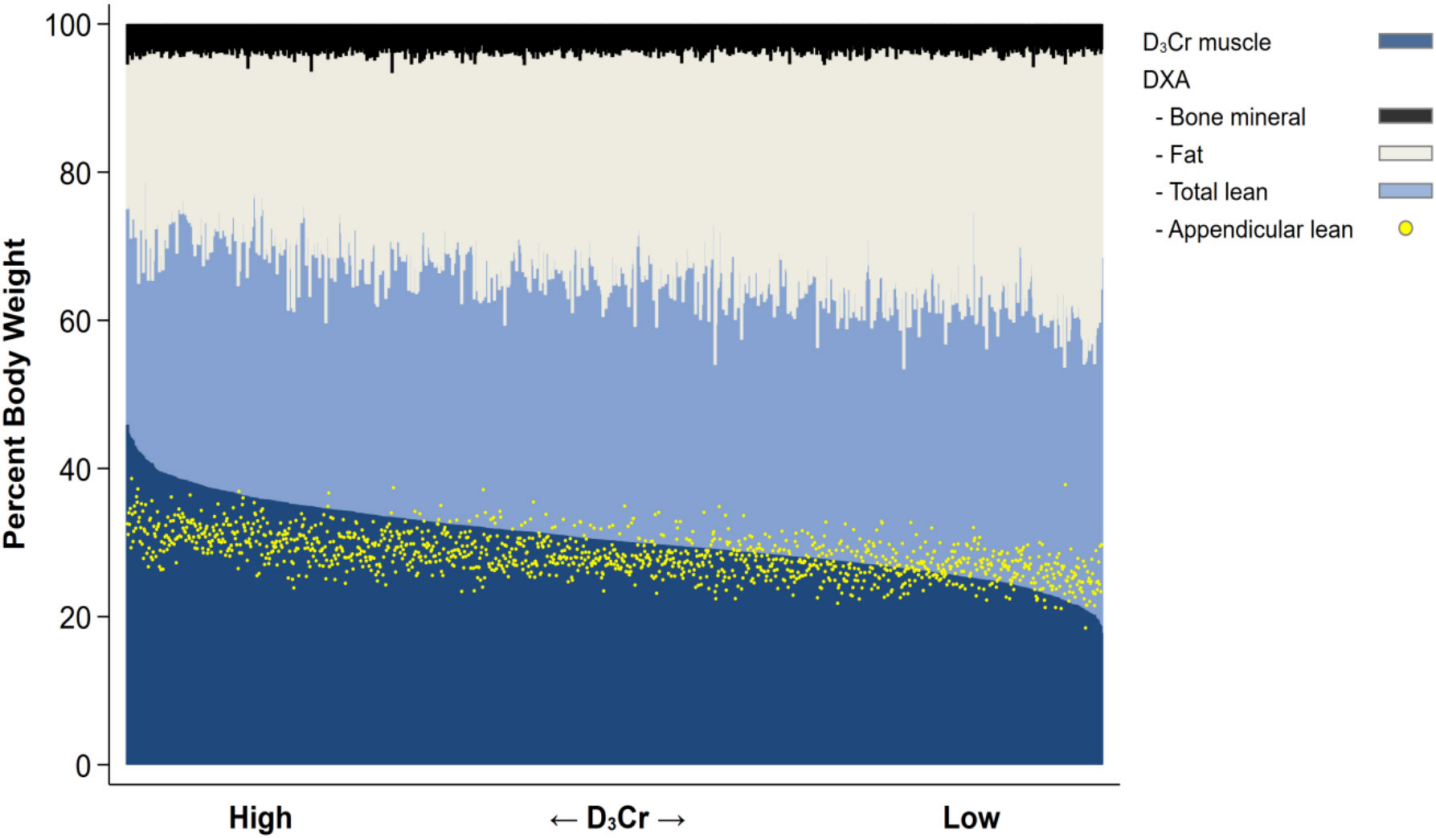
Body composition of men > 80 years in the MrOS cohort, *n* = 1300.

Innovations in measurement techniques embrace the D_3_‐creatine dilution method (D_3_Cr) that is much advocated by Bill Evans. This novel, non‐invasive technique uses stable‐isotope labelled creatine to measures total muscle mass via a single urine sample, providing an accessible and scalable diagnostic tool. Evans explained that the D_3_‐creatine dilution method is “a method by which we give deuterated creatine. The beauty of creatine is that it is not synthesized in muscle, it is synthesized in the liver and kidney, so a small tracer dose of deuterated creatine is ingested and transported to all muscle in the body. The deuterated creatine is converted to creatinine, a waste product without biological function, and then rapidly excreted into urine. We can take a single spot urine sample at steady state and look at the enrichment of the creatinine molecule, determine what the creatine pool size is, and from that, we can calculate muscle mass”. Evans explained that creatine is present in skeletal and cardiac, but not in smooth muscle. He estimated the time after ingestion to reach isotopic steady state to 48 to 96 h and explained that validation studies have demonstrated the accuracy and practicality, with applications spanning both clinical practice and large‐scale research studies. According to Evans, the D_3_‐creatine dilution method's scalability and efficiency make it particularly promising for addressing diagnostic gaps as in contrast to previous studies that were based on measures of DEXA appendicular lean mass, the results by Evans's group using the D_3_Cr dilution method suggest that “muscle mass is a primary determinate of physical performance and adverse outcomes, and that the relative effects of higher body fatness are less important”. By offering reliable and reproducible muscle mass assessments, this method could facilitate the development of standardized diagnostic criteria and improve cross‐study comparability. Accordingly, Evans highlighted the difficulties in correctly analysing DEXA data. Showing the data in Figure 1, he said that these are “from a population study that we did where we used both DEXA measurement and D_3_Cr. What you can see is (that) muscle mass is lined up in these older men, (…) 80 years and older, more than 1300 men from the MrOS study cohort, from high muscle mass down to low muscle mass. Dark blue is muscle, light blue is the other stuff that's in lean body mass. Dark plus light blue is lean body mass. You can see that the relationship is highly, highly variable, that yellow is appendicular mass for which we saw no relationship”.

Prof. Evans pointed out that several studies have shown that “D_3_Cr muscle mass was the only body composition variable that co‐segregated with strength and physical performance measures, and contributed to a factor that was associated with disability outcomes in older men” in one study [9] and that the findings of another study [10] “support efforts to evaluate the D_3_Cr dilution method in clinical applications including in future definitions of sarcopenia”.

Calling for action, Evans said that there is a lack of public advocacy and that there is little public awareness of how sarcopenia affects the risk of disability: “Health care professionals believe that sarcopenia is a natural consequence of aging, and there has been inaction by FDA and EMA”. What is actually needed, Evans said, is that “sarcopenia should be defined as low skeletal muscle mass undiluted by fibrosis, lipid, connective tissue”. However, this proposition sparked debate. Prof. Heymsfield raised an important question: What remains of skeletal muscle mass if fibrosis (connective tissue), lipids, and other components are excluded? Indeed, muscle mass, as typically measured by CT and MRI, inherently includes these elements, such as connective tissue, lipids, and blood. To refine this concept, it could be suggested that D_3_ dilution space offers a specific estimate of myofiber mass. However, at present, no established model exists to directly relate D_3_ dilution space to myofiber mass, leaving this interpretation as an area requiring further investigation.

### 1.3 Discussion

During the discussion, a heated debate was triggered. One of the questions raised was whether the D_3_‐creatine method is scalable for real‐world applications, given that only a limited number of laboratories are equipped to perform mass isotopomer distribution analysis (MIDA) by mass spectrometry, and 24‐h urine collection poses significant challenges for clinicians. Besides, cost, turnaround time, and related clinical factors make it difficult to implement D_3_Cr at a practical level. It may be possible to measure body fat for obesity with deuterium dilution, but an approach identical to D_3_ would be considered implausible in clinical settings. In addition, it was discussed that D_3_ dilution does not quantify “muscle mass”, rather it estimates creatine dilution space. The dilution space “correlates” with muscle mass and that the association gives rise to prediction models for estimating muscle mass. Prof. Steven Heymsfield (Pennington Biomedical Research Center, Baton Rouge, Louisianna, United States) explained that we do not have enough data at present to establish if those models are applicable across different populations, age groups, etc. At the moment, he explained, we know that the current simple model (D_3_ dilution space) does not apply in some groups, and much more research efforts are needed in larger, more diverse populations.

Evans explained that the method does not require 24‐h urine collection or single‐spot urine tests. In a study involving 11 000 women over the age of 80, all procedures were conducted remotely. Participants were mailed the D_3_‐creatine capsule and subsequently returned their urine samples for analysis. The analytical method employed is a straightforward high‐performance liquid chromatography (HPLC) technique, which measures creatinine enrichment. This approach eliminates the need for MIDA and allows the analysis to be performed by any hospital core laboratory, making the method scalable to large populations.

A discussion was initiated on the potential implications of adopting a simplified definition of sarcopenia based solely on muscle mass. Concerns were raised that such a definition might create challenges, particularly when considering its alignment with regulatory standards. Specifically, if the definition relies on outcomes that are not recognized as approvable endpoints by regulatory authorities, it may inadvertently steer the field into a restrictive position. This could result in a scenario where companies focus on muscle mass as the primary endpoint for sarcopenia interventions. However, regulatory agencies may not accept muscle mass as a clinically meaningful endpoint, requiring alternative measures instead. Such misalignment could complicate the development of therapies and hinder progress in the field. A more effective approach might involve defining sarcopenia in terms of clinically meaningful outcomes such as muscle function and mobility, ensuring alignment with regulatory expectations and improving the translational impact of research and interventions.

A point of clarification was raised regarding whether the definition presented aligns more closely with myopenia, a concept that has previously been discussed but did not gain traction as a term. Evans replied that whilst myopenia represents a general loss of muscle, sarcopenia is the age‐related loss of muscle and function [11]. Therefore, sarcopenia, traditionally defined as lower muscle mass specifically associated with aging, differs from this broader concept of muscle wasting. Recent developments were noted in advancing muscle mass measurement as a diagnostic outcome. A second meeting with the FDA is forthcoming, and approval for a diagnostic method to measure muscle mass is anticipated. This will enable clinicians to prescribe muscle mass testing for patients using an FDA‐approved outcome measure. While this progress does not yet establish a definitive criterion for sarcopenia, the method has been integrated into several large‐scale studies, including the Framingham Heart Study, the Women's Health Initiative (WHI), the Study of Muscle, Mobility, and Aging (SOMMA), and the Tobago Longitudinal Aging Study. Data from these studies indicate clear associations between muscle mass and mobility disability risk. For men over 60 years of age, muscle mass below 25% is strongly associated with a significantly increased risk of mobility disability. Among women, a similar risk is observed when muscle mass falls below 20%, approximately the 10th percentile of the population. These findings, based on published data, underscore the potential of muscle mass thresholds to predict mobility‐related outcomes, although further research is needed to establish definitive diagnostic criteria for sarcopenia and determine precise cut points.

A discussion was also raised regarding the reliance on muscle mass as a predictive measure, particularly in contexts such as glucose tolerance and energy expenditure. It was noted that reducing muscle mass while proportionally increasing insulin sensitivity may have a negligible impact on glucose tolerance. Similarly, while muscle constitutes a smaller portion of resting metabolic rate, it accounts for most individual differences in resting metabolic rate. The physiological functions of muscle, including its role in glucose tolerance, body weight regulation, longevity, and cardiovascular disease risk, were highlighted as critical considerations for patients with sarcopenia. Muscle mass alone may not predict all clinically relevant outcomes in sarcopenia. Instead, sarcopenia has been defined based on functional outcomes which demonstrate a strong correlation with muscle mass, illustrating the potential benefit of a clearer distinction between “sarcopenia” and “dynapenia” [12]. Recent findings indicate that muscle is independently associated with glycaemic control. Moreover, muscle mass and muscle protein synthesis rates are fundamental determinants of changes in metabolic rate associated with weight gain and weight loss. The extent of muscle involvement depends on the specific outcome of interest, and its contribution is not absolute. In comparing dystrophy to age‐related muscle changes, it was emphasized that these processes are distinct. Studies profiling aged muscle across the lifespan in rats, now being extended to humans, reveal differences between dystrophy and sarcopenia. Dystrophies are hereditary diseases characterized by progressive muscle fibre degeneration, whereas sarcopenia appears primarily driven by a dramatic increase in inflammatory signalling, including interferon pathways, resembling responses to viral infections. This is thought to result from the transcription of long repetitive elements in aging. Additionally, sarcopenia is associated with neuromuscular junction remodelling and regression of motor neurons, changes that are unique to aging and absent in typical atrophy observed in younger individuals. The distinction between age‐related sarcopenia and muscle atrophy in younger adults is critical. While a simplified definition of sarcopenia may emphasize muscle mass loss, it is important to recognize the unique challenges and mechanisms associated with aging. Conflating muscle loss in a 30‐year‐old with that in an 85‐year‐old overlooks these age‐specific factors and the complexity of sarcopenia in older populations.

One question finally concerned whether muscle changes associated with aging, including intramyocellular and intermuscular adipose accumulation, parallel those observed in rapidly progressive diseases such as Duchenne muscular dystrophy, where a transition from intramuscular fat (i.e. intramuscular adipose tissue) to fibrosis occurs. Evans pointed out that a recent multi‐centre study on Duchenne muscular dystrophy, which included over 100 patients aged 4 to 24, demonstrated that when muscle mass declines to approximately 20%, individuals lose the ability to walk, irrespective of age [13]. This finding underscores the critical threshold for functional mobility in this condition. The use of GLP‐1 receptor agonists is increasingly adopted among paediatric neurologists treating Duchenne muscular dystrophy due to significant weight gain observed in these patients. Unfortunately, data on the effect of these drugs on muscle mass and function in dystrophy and sarcopenia are not available yet. Extensive fibrosis is also a prominent feature of the disease. While the progression of fibrosis and fat infiltration in aging is more gradual, both conditions exhibit common pathological characteristics, such as increased fibrosis and intramuscular fat deposition. In Duchenne muscular dystrophy, these changes occur at a markedly accelerated pace.

## 2 Trial Endpoints for Sarcopenia in Ageing

The discussion around endpoints in clinical trials on sarcopenia was led by Prof. Roger A. Fielding (Tufts University, Boston, Massachusetts, United States). He started out saying that the evaluation of sarcopenia, particularly in aging populations, requires carefully defined and clinically meaningful endpoints to ensure effective trial designs and regulatory approvals. Fielding explained that “You ask older people, ‘Can you walk to a city block, or can you climb a flight of stairs?’ If a patient has a mobility disability, we do the test, and we can determine whether their mobility disability is due to low muscle mass or some other reason”. Sarcopenia is “a diagnosis of exclusion of other causes, and that's what FDA, the devices people like. If you have a higher muscle mass, mobility disability is probably due to a neurological problem, balance, eyesight, many other problems that they can treat; if it's low muscle mass, unfortunately, we don't have a drug yet to treat it. We hope that we will”.

Objective measures of muscle mass are crucial to understanding who should be enrolled in clinical trials. These measures also help identify patients whose mobility issues stem from low muscle mass, making them ideal candidates for interventions aimed at increasing muscle mass or function. Data from census studies highlight the prevalence of mobility‐related issues among older adults. Among individuals aged 65 and older living in community settings, 39% report difficulty walking or climbing stairs. A significant proportion of these individuals are unable to walk 400 m or climb a flight of stairs. These statistics underscore the strong association between muscle loss and reduced mobility in aging populations. Therapies targeting sarcopenia or muscle dysfunction must address specific pathologies, such as motor unit loss, fibre type changes, muscle atrophy, and neuromuscular junction alterations as well as the age‐associated decrease of force transfer between muscle, connective tissue, and bone [14]. These impairments affect key physiological properties of muscle, including strength, power, and fatigue resistance. Phase I and II studies often focus on these metrics as primary outcomes to assess the efficacy of therapeutic interventions.

Validated measures of physical performance—such as the Short Physical Performance Battery (SPPB), habitual gait speed tests, stair‐climb tests, and the 6‐min walk test—offer valuable insights into functional capacity. These measures are particularly relevant in phase III trials, where the focus shifts to functional limitations and disability outcomes. Additionally, remote sensing and wearable technologies hold promise for future applications in assessing real‐world physical function and monitoring intervention impacts. Muscle performance in older adults operates within a scaled‐down version of the force‐velocity curve. Therapeutic interventions targeting muscle function should consider the distinct physiological properties of muscle, including strength, power, fatigue resistance, and endurance. While these properties are interrelated, they represent distinct domains that require precise evaluation in clinical trials.

Fielding provided an overview of the several well‐validated and reliable measures of physical performance as well as their respective meaningful changes that may serve as key endpoints in sarcopenia trials:

- *Short Physical Performance Battery (SPPB)*: A composite measure predictive of disability, hospitalization, and mortality. Clinically meaningful improvements (MCID) in SPPB scores have been demonstrated in interventions such as the Lifestyle Interventions and Independence for Elders (LIFE) study and estimated at 0.4–1.5 points [15]
- *Habitual Gait Speed*: An indicator of overall mobility and functional status (MCID 0.05–0.1 m/s)
- *400‐Meter Walk and Six‐Minute Walk Tests*: Assessments of endurance and walking capacity (MCID 400‐m walk: 50–60 s [10], 6‐min walk test: 25–50 m [16])
- *Stair‐Climbing Tasks*: Measures of lower‐limb strength and power MCID: 2–4 s [17])

Fielding elaborated that careful training of study personnel ensures the reliability and sensitivity of these measures, enabling accurate detection of intervention effects. He explained that “What we're trying to do with these measures of capacity that we've used is really trying to understand how older individuals live and perform on their own within their own environmental constraints. That's where we try to look and transition into assessing disability status and how that's related to falls, fractures, death and other real, hard clinical outcomes. I would argue that that these domains of functional limitation and disability really can be considered or can be strongly considered for outcomes in phase‐three trials”.

The LIFE study [18], involving 1600 older adults across eight clinical centres, demonstrated that physical activity interventions significantly improved physical function and delayed the onset of major mobility disability. [19] Fielding said that “What's interesting here (from the parameters of the SPPB) that it's actually the chair‐stand test that really is driving the improvement in physical function, at least that's what we see with a physical activity intervention”. He further explained that “In the main study, we had selected the primary outcome of interest to be the onset of major mobility disability. This is another attractive outcome that could be used in trials, and this is really the development of the inability to walk 400 meters. At baseline, everybody has their 400‐meter walk measured. They have to be able to complete a 400‐meter walk within 15 minutes, and then they are reassessed. In this case, it was every six months during the intervention, and they had the outcome when they could no longer complete the 400‐meter walk”. Similar results were observed in the SPRINTT trial, a European study that replicated the findings using multi‐component interventions combining nutrition and physical activity [20]. These trials underscore the potential for interventions to reduce the incidence of mobility disability and highlight the importance of using validated physical performance measures as endpoints. Fielding said that “regardless of how we define sarcopenia, we really have endpoints that are related to changes in physical functioning”.

Recent advances in remote sensing and wearable technologies offer exciting possibilities for real‐world assessments of physical activity and mobility. For example, the EMA‐approved SV95C gait‐monitoring sensor has been used as a digital endpoint in Duchenne muscular dystrophy trials [21]. However, challenges remain in applying similar technologies to older adults with low physical activity levels, where distinct activity features are difficult to discern. Despite these challenges, the future integration of such technologies holds significant potential for sarcopenia trials. But, as Fielding pointed out, the problem “using accelerometries in older adults that have poor physical functioning and probably have sarcopenia, is that the activity signal that you get from those individuals is very low. They spend most of their days sitting, walking very slowly, walking short bursts of activity, and to pick up distinct features from those signals represents an analytical challenge”.

Fielding concluded by saying that several factors have hindered the development of approved pharmacological therapies for sarcopenia:

1. *Trial design issues*: Standardization of performance outcomes across multiple trial sites remains a challenge.
2. *Lack of combined interventions*: Combining drug therapies with exercise or behavioural interventions may enhance physical function but poses operational challenges.
3. *Regulatory barriers*: High standards for demonstrating clinically meaningful outcomes are necessary to ensure the safety and efficacy of treatments for sarcopenia.
4. *Consensus on definitions and endpoints*: Ongoing debates about the definition of sarcopenia and appropriate endpoints need resolution to streamline trial designs and regulatory approvals.
5. *Patient‐reported outcomes (PROs)*: The development of specific PROs for sarcopenia will enhance the assessment of treatment impacts on quality of life and daily functioning.

Fielding's conclusion was that sarcopenia trials in ageing populations must prioritize clinically meaningful endpoints that reflect improvements in physical function and quality of life. Validated performance metrics, remote sensing technologies, and combined therapeutic approaches represent critical components of future trial designs. Addressing current challenges, including standardization and regulatory hurdles, will pave the way for the successful development and approval of therapies targeting sarcopenia in older adults.

Overall, it was made clear during the meeting that from a regulatory treatment development standpoint, clinically meaningful changes in PROs, physical function and/or morbidity/mortality need to be shown. If the latter is not an efficacy endpoint, safety needs to documented. Given that the population that may be addressed in the aging associated sarcopenia is vast, the safety requirement standards applied for studies may be equivalent to those of studies in T2DM. Some argued at the meeting that, that this would make study programs so large that from an economic standpoint only therapies that significantly impact on morbidity/mortality outcomes have a chance to be considered commercially feasible for development.

### 2.1 Discussion

A long discussion ensued after Prof. Fielding's presentation with the participants of the workshop being much involved. Indeed, aiming for harder endpoints in sarcopenia trials presents challenges related to trial size and duration. However, when one talks to older individuals, their primary concern is not usually the chronic conditions they live with but rather the loss of mobility and independence. These outcomes are critically important and therefore deserve attention. How we measure and define these as endpoints is a key discussion point. While there is room for debate about the best approach, mobility is undeniably a significant issue for many older adults. In this context, it is also worth noting that the choice of endpoints will often depend on the mechanism of action of the drug being tested. For example, endpoints like the SPPB or chair stand can suffer from ceiling effects, which may limit their usefulness in certain patient populations. Stair climb power, on the other hand, has been successfully used in FDA trials for cachexia, particularly by experts like Mitch Steiner [22]. It is a promising endpoint but still depends on the drug's specific mechanism of action. Hard endpoints, on the other hand, need to align with the drug's effects. For example, a drug that impacts muscle mass might better influence strength. Yet, using force production as an endpoint remains contentious, as regulators have not classified it as a functional outcome.

Understanding how muscle strength contributes to meaningful functional outcomes is critical. For patients who score highly on tests like SPPB or gait speed, we might question the necessity of treatment, but reaching consensus on what constitutes a clinically meaningful difference is essential. Responder analyses—i.e. proportions of patients achieving clinically meaningful changes at varying levels—are crucial, given the variability in tests like the 25‐ to 50‐m walk. Such different approaches, including anchoring outcomes, have informed some of these metrics. The discussions about sarcopenia and cancer cachexia populations being distinct yet overlapping are important. Most cancer patients are older, which complicates the interplay of multimodal contributors to cachexia. Going forward, aligning definitions of sarcopenia and cachexia and identifying endpoints applicable across both conditions could be valuable. PROs should not be overlooked. Metrics like the ability to perform daily activities (e.g. going to the bathroom, shopping, or leaving the house) could serve as hard endpoints. However, these require addressing patient confidence, especially for those who have experienced falls. Rebuilding confidence and ensuring sufficient study durations to capture functional gains post‐muscle restoration are additional challenges. Short‐term studies may miss these subtleties.

In much of the ensuing discussion, comments on health span were widely acknowledged. Indeed, many patients express a desire to avoid dependency rather than focus solely on extending lifespan. While observational studies have examined functional decline leading to death, few intervention studies exist. This is an area worthy of exploration. The observation about defining clinically significant endpoints is notable as it parallels challenges in designing non‐inferiority trials, where judgement is key. Learning from such trials could provide insights into setting meaningful thresholds for functional improvement.

When considering mobility, actigraphy offers promise but comes with practical limitations. Devices must be worn continuously to capture critical incidents like nighttime falls. While wearable technology can provide objective data, compliance remains a challenge. Operational considerations, such as device placement (e.g. wrist vs. pendant), also influence outcomes. Finally, the economic and logistical hurdles of developing treatments for conditions like sarcopenia cannot be ignored. Mary Parks, formerly working for the FDA, noted that proving safety for sarcopenia might require 7000 patient‐years of data, a daunting prospect for any development program. Hard outcomes such as fall‐free survival or reduced hospitalization may be more feasible in defined subpopulations, like those with sarcopenia and heart failure or cancer.

## 3 Treatments for Sarcopenia in Ageing and Regulatory Issues

Prof. Vickie Baracos (University of Alberta, Canada) opened the session on therapeutic approaches to counteract sarcopenia by sharing her views from a focused perspective developed through the measurement of lumbar skeletal muscle index (LSMI) in about 25 000 cancer patients. She said that the primary objective of these assessments has been to evaluate whether muscle mass is high or low, to identify appropriate interventions, or to recognize when the opportunity for addressing sarcopenia may have passed. For Baracos, one of the most striking findings is the immense variability in LSMI values among cancer patients. In a cohort with a median age of 65 years, male patients demonstrate a range from 25 to 80 cm^2^/m^2^. This variability is substantial, with the lowest value being less than one‐third of the highest. She added that the question of what constitutes “normal” muscle mass has gained attention in recent years. Current estimates of normality are often derived from healthy individuals in their 30s, such as kidney donors. For example, the mean LSMI for 30‐year‐old men is approximately 60 cm^2^/m^2^, a value that aligns with the 75th percentile of older cancer patients. Remarkably, some cancer patients exhibit LSMI values equal to or exceeding this benchmark, indicating a subset of individuals who are not sarcopenic and may not benefit from sarcopenia‐targeted interventions. In the recent phase 2 trial of the growth differentiation factor (GDF) 15 antibody ponsegromab vs. placebo [23] in patients with cancer cachexia due to non–small‐cell lung cancer, pancreatic cancer, or colorectal cancer, there was a 5.0% increase in LSMI at week 12 in the subgroup that received 400 mg ponsegromab. In the ROMANA 1 and 2 trials, comparing anamorelin vs. placebo in patients with non–small‐cell lung cancer, the increase in LSMI reached about 3.2% with anamorelin at 12 weeks of follow‐up [24]. Baracos, however, also highlighted the sources of variation LSMI assessed at L3:

- Variation at instrument or imaging level
- Reader precision
- Biological/clinical variation due to
  ○ Individual variability
  ○ Categorical versus continuous
  ○ Effects of cancer therapy
  ○ Effects of tumour response

Precision in this context is determined by reading 30 images × 2 or 15 images × 3 with the least significant change being the smallest difference that can be detected above measurement error [25]. Biological variation, on the other hand, can be extensive among patients with identical diagnoses, stages, or treatment plans [26].

Baracos continued by saying that a thorough understanding of the development of sarcopenia in cancer patients reveals a multifaceted trajectory. Diagnostic imaging records offer a longitudinal perspective, tracing sarcopenia onset to prior health events, sometimes years or decades earlier. For instance, orthopaedic conditions, prolonged surgical waiting times, or complications can initiate declines in muscle mass. Thus, sarcopenia often arises from a complex interplay of health conditions. For example, patients with non‐small‐cell lung cancer frequently present with comorbid chronic obstructive pulmonary disease (COPD), which accelerates muscle loss beyond the rates associated with normal aging. This highlights the interplay between chronic diseases and muscle degradation.

A critical consideration in this context is whether sarcopenia represents a slow, decades‐long decline or is predominantly driven by acute episodes. Chronic, gradual muscle loss may necessitate long‐term pharmacological interventions. However, emerging evidence suggests that significant muscle degradation often occurs during acute events, such as hospitalizations, ICU stays, or postoperative complications. Baracos noted that in cancer care, systemic therapy is a prominent driver of acute muscle loss. Chemotherapy, immunotherapy, and targeted therapies are associated with a 5–14% reduction in total muscle mass within 100 days—a loss equivalent to approximately 25 years of natural aging in a healthy individual. These profound iatrogenic losses demand a paradigm shift in the approach to sarcopenia management. Instead of conceptualizing sarcopenia solely as a chronic condition, there is a need to address the acute and substantial muscle damage induced by antineoplastic therapies. This could involve recognizing these treatments as contributors to muscle loss and developing strategies to mitigate their impact during critical treatment phases. By prioritizing interventions at these junctures, she said, we can better address the acute dimensions of sarcopenia and improve patient outcomes in cancer care.

Prof. Stephan von Haehling (University Göttingen Medical Center, Germany) then presented an overview of anabolic agents and other supplements to be used in patients with muscle wasting. He started with a brief discussion of muscle anabolic drugs in the context of heart failure that reveals a range of therapeutic approaches, each with unique outcomes and limitations. Indeed, testosterone has been extensively studied in smaller heart failure trials, demonstrating notable efficacy. In a prominent example was reported by Rosano et al. [27], which included 70 symptomatic male heart failure patients (median age 70 years; NYHA class II and III). Over a 3‐month period, testosterone treatment led to an increase of 80 m in the 6‐min walk test distance—a remarkably significant improvement rarely observed in major trials. Additionally, the trial reported an increase in peak oxygen consumption of 3 mL/kg/min, further emphasizing the drug's impact on physical performance.

Other drugs used in clinical trials showed mixed results [28]: Enobosarm (a selective androgen receptor modulator, SARM) applied vs. placebo in the POWER trials yielded inconsistent signals for improvements in muscle strength despite increases in muscle mass [29]. Anamorelin used vs. placebo in the ROMANA trials significantly increased median lean body mass but failed to enhance functional metrics such as hand‐grip strength [24]. A recent phase 2 trial using ponsegromab vs. placebo in patients with cancer cachexia reported notable increases in body weight at higher doses, though data on changes in fat mass and corresponding functional improvements remain lacking [23]. These findings, von Haehling pointed out, highlight a recurring issue: Increases in lean muscle mass often fail to translate into corresponding gains in muscle strength, limiting the functional efficacy of these interventions.

Nutritional supplementation has shown promise in improving muscle mass and function in specific patient populations. A trial conducted with Rozentryt et al. involving 29 patients with cardiac cachexia demonstrated that providing an additional 600 cal per day increased both lean tissue and body weight over 6 weeks [30]. An investigator‐blinded trial with 38 clinically stable heart failure patients tested essential amino acid supplementation (predominantly branched‐chain amino acids). Results showed significant improvements in both 6‐minute walk test distance and peak oxygen consumption measured on a treadmill [31]. Finally, iron supplementation offers benefits beyond treating anaemia, particularly in heart failure patients. Iron supports mitochondrial function, as key enzymes in the respiratory chain are iron‐dependent. A recent study utilized magnetic resonance spectroscopy to demonstrate the effects of iron on muscle bioenergetics [32]. Results indicated that treating iron deficiency reduced the half‐life of phosphocreatine, facilitating faster ATP generation for muscle contraction. This underscores the broader physiological benefits of iron in improving muscle energy metabolism.

Taken together, von Haehling concluded that despite the variety of approaches, a critical challenge remains: translating increases in lean muscle mass into functional improvements in strength and performance. Future research needs to prioritize interventions that directly address this gap, particularly in the context of acute and chronic muscle wasting conditions.

Dr. Waly Dioh, Chief Clinical Operations Officer at Biophytis Inc. presented the therapeutic approach of the company that is advancing the development of 20‐Hydroxyecdysone (20E) [33], a drug candidate from the steroid hormone class, targeting sarcopenia and frailty [34]. The development pathway includes both phase 2 and phase 3 trials, leveraging insights from earlier observational studies and aligning clinical endpoints with patient‐centric outcomes.

An initial interventional phase 2 study (NCT03452488) utilized the 400‐m gait speed as the primary endpoint. This study confirmed the natural deterioration in gait speed observed in the earlier SARA‐OBS observational study (placebo group). At the highest dose of 350 mg twice daily, treatment with 20E demonstrated a nearly significant improvement in gait speed, supported by evidence of a dose–response effect. Secondary mobility assessments, including the six‐minute walk distance and four‐meter gait speed, also revealed positive trends. Subgroup analyses further highlighted the efficacy of 20E in specific high‐risk populations, such as patients with sarcopenic obesity. In this subgroup, improvements in the 400‐m gait speed were accompanied by significant outcomes in other functional measures, including the chair stand test and assessments of slow walkers. These findings identify high‐risk populations as optimal targets for 20E treatment.

Building on the promising phase 2 results, the phase 3 study shifts focus from physical performance metrics to major mobility disability (MMD) as the primary endpoint. MMD, defined as the inability to complete a 400‐m walk within 15 min, represents a critical milestone in the progression of sarcopenia. It serves as a meaningful and clinically relevant outcome, closely associated with adverse events such as falls, fractures, hospitalizations, and mortality. The trial design incorporates lessons from the SPRINT and LIFE studies discussed above, aiming to enrol 900 patients with severe sarcopenia, aged 65 or older, who will be treated for at least 12 months. Key eligibility criteria include: (i) living independently in the community, (ii) evidence of motor function loss within the past year, (iii) low SPPB scores, (iv) low hand grip strength, (v) low gait speed.

Dr Dioh noted that the phase 3 study design has received endorsement from major regulatory agencies, including the FDA and European bodies, with ongoing discussions with different Ethics Committee. Biophytis is committed to advancing 20E as a potential treatment for sarcopenia, addressing critical gaps in clinical care through rigorous and meaningful evaluation.

Dr. David J. Glass of Regeneron Inc. went on to discuss emerging muscle building drugs and approaches. He pointed out that he sees two primary pathways that regulate muscle mass:

1. Inhibition of the myostatin‐activin signalling pathway
2. Activation of Akt signalling

These pathways converge largely through insulin‐like growth factor 1 (IGF‐1) signalling, which plays a pivotal role in muscle hypertrophy. For instance, localized production of IGF‐1 in muscle during anabolic exercise drives hypertrophy, as seen in targeted strength training. Additionally, inhibiting myostatin enhances muscle growth, making it a key target for therapeutic intervention. Myostatin (GDF‐8), a member of the transforming growth factor‐beta (TGF‐β) superfamily, was first described by Lee's group in 1997 [35]. His work showed that animals with myostatin deficiencies, such as Belgian Blue cattle and certain whippets, exhibit remarkable increases in muscle mass. These findings highlight the critical role of myostatin in limiting muscle growth and underscore its therapeutic potential. However, in humans, Glass pointed out, the effects of myostatin inhibition alone are modest. Anti‐myostatin antibodies increase muscle mass by only ~2% in humans, compared to 15% in mice. This discrepancy likely arises from species‐specific differences in additional ligands, such as activin A, which also signal through the activin type 2 receptor (ActRIIB). Blocking both myostatin and activin A in humans results in a 6–8% increase in skeletal muscle mass, comparable to directly inhibiting ActRIIB.

Importantly, ActRIIB signalling also involves ligands like GDF‐11, a “super myostatin” whose levels increase with age in skeletal muscle. This age‐related upregulation suggests ActRIIB inhibition may be particularly beneficial for older populations. A study at Novartis demonstrated that using the ActRIIB antibody bimagrumab led to a 6–8% increase in muscle mass within a month of a single dose [36]. Remarkably, this also reduced fat mass, even without caloric restriction or GLP‐1 agonists, likely due to the higher metabolic activity of increased muscle tissue.

The second major pathway, as discussed by Glass, involves Akt signalling, primarily activated via IGF‐1. Akt promotes muscle hypertrophy by stimulating protein synthesis through the mTOR pathway and by suppressing muscle breakdown by inhibiting E3 ubiquitin ligases. Chronic inflammation, common in aging, activates E3 ligases, contributing to muscle loss. Thus, anti‐inflammatory strategies may complement Akt‐targeted therapies to combat sarcopenia. Therapeutic activation of Akt has shown dramatic results in preclinical models, with significant muscle hypertrophy observed in mice. However, uncontrolled Akt activation can lead to dystrophic muscles due to unchecked growth. IGF‐1, with its built‐in regulatory mechanisms, provides a safer route to modulating Akt activity. Testosterone, a classic anabolic agent, also promotes muscle growth partly through IGF‐1 signalling.

Several ligands, including myostatin, activin A, and GDF‐11, feed into the ActRIIB and Akt pathways, making them key targets for sarcopenia therapies. The balance between muscle hypertrophy and the risk of adverse effects, such as dystrophic changes, remains critical. For cachexia, interventions that increase muscle mass without caloric changes also induce fat loss, offering additional benefits for metabolic health. However, given the positive relationship between body fat and survival in patients with cancer cachexia, metabolic benefits of lipolysis will have to be carefully balanced with potential drawbacks.

Glass summarized, whilst significant progress has been made in understanding the mechanisms of muscle hypertrophy, ongoing research needs to address the complexities of these signalling pathways. By carefully targeting these mechanisms, therapies can effectively increase muscle mass and strength, providing meaningful benefits for individuals with sarcopenia or cachexia.

### 3.1 Discussion

When asked about the use of PI3 kinase inhibitors for treating cancer and their effects on muscle mass, Glass answered that PI3 kinase (PI3K) inhibitors, which are widely used in oncology, present a potential challenge to skeletal muscle maintenance. These inhibitors can interfere with the Akt/mTOR signalling pathway, a key driver of muscle hypertrophy. Chronic suppression of mTOR signalling through agents like rapamycin has been shown to block muscle hypertrophy. Despite this, there is a paradoxical increase in mTOR activity observed with aging. This heightened activity may contribute to age‐related muscle degeneration by promoting the accumulation of misfolded proteins. As such, blocking mTOR activity through rapamycin during aging has shown benefits regarding the maintenance of muscle mass and strength in rodents. Glass added that current research is exploring whether intermittent (pulsed) mTOR inhibition might facilitate autophagy without impairing muscle growth, though this remains a complex and nuanced area of investigation.

A second question was asked concerning the lack of addressing the stem cell niche and the role of stem cells in aging and muscle mass and whether Glass thinks that this poses a significant contributor as well? Glass replied that Satellite cells, the resident stem cells in muscle tissue, play a critical role in maintaining and repairing skeletal muscle. Ageing disrupts the functionality of these cells, partly due to chronic inflammatory signalling that prematurely activates them. Once activated, satellite cells often fail to differentiate terminally, leading to the replacement of muscle tissue with fat and fibrosis. This process inhibits muscle hypertrophy and exacerbates age‐related declines in muscle function. Despite these challenges, exercise remains an effective intervention for preserving and restoring muscle mass in older individuals. For instance, Glass explained, aerobic exercise has been shown to improve neuromuscular junction function and muscle performance even in individuals in their 90s. Developing therapies that mimic or enhance the effects of exercise could offer promising avenues for treating sarcopenia.

## 4 Conclusions

The December 2024 regulatory workshop in Washington D.C. addressed challenges in diagnosing sarcopenia, focusing on the limitations of early definitions based on DEXA‐derived muscle mass measurements and emphasizing the stronger correlation between muscle strength, functional performance, and clinical outcomes. The inconsistent use of terms such as “sarcopenia” (loss of muscle mass and function), “dynapenia” (loss of muscle strength), and “myopenia” (primarily loss of muscle mass) further complicates the field and hinders progress. A meta‐analysis highlighted significant inconsistencies in prevalence estimates, driven by varying diagnostic approaches, and called for standardized criteria. One promising solution may be assessing muscle mass relative to body weight, while the D_3_‐creatine dilution method, championed by Bill Evans, may offer a potentially scalable, non‐invasive tool for accurate muscle mass measurement, bridging diagnostic gaps and enhancing cross‐study comparability. The ensuing discussions underscored the potential of the D_3_‐creatine dilution method in sarcopenia assessment, while also raising concerns about its scalability, regulatory acceptance, and the need for uniform diagnostic criteria. Key debates focused on differentiating sarcopenia from general muscle loss, the relative importance of muscle mass versus function in defining the condition, and its broader implications for clinical practice and metabolic health.

The workshop also delved into the design of clinical trials for sarcopenia, emphasizing the need to prioritize clinically meaningful endpoints, such as physical function and quality of life, to ensure that interventions are truly effective. Participants agreed that addressing standardization, regulatory challenges, and the integration of emerging technologies will be crucial for advancing treatment development. The subsequent discussion highlighted the difficulties in defining meaningful endpoints for sarcopenia trials, especially when balancing trial feasibility with the necessity of clinically relevant outcomes like mobility and independence. Attendees stressed the importance of incorporating patient‐reported outcomes, acknowledged the limitations of certain functional tests, and discussed the regulatory and logistical challenges involved in developing effective treatments. Therapeutic approaches to sarcopenia, highlighting advancements in muscle mass interventions, include 20‐hydroxyecdysone (20E) and anabolic agents like testosterone and myostatin inhibitors. Experts emphasized the need for treatments that not only increase muscle mass but also improve muscle strength and functional outcomes, particularly for cancer and heart failure patients experiencing muscle wasting.

From a regulatory treatment development standpoint, clinically meaningful changes in patient reported outcomes, physical function and/or morbidity/mortality are urgently needed. If the latter is not an efficacy endpoint, safety needs to be documented. Given that the population that may be addressed in aging associated sarcopenia is vast, the safety requirement standards applied for studies may be equivalent to those of studies in type 2 diabetes mellitus. Concern remained that such an approach would make study programs so large that from an economic standpoint only therapies that significantly impact on morbidity/mortality outcomes have a chance to be considered commercially feasible for development.

## References

[1] M. Critchley , “The Neurology of Old Age,” Lancet 217 (1931): 1331–1337.

[2] I. H. Rosenberg , “Sarcopenia: Origins and Clinical Relevance,” Journal of Nutrition 127 (1997): 990S–991S. 9164280 10.1093/jn/127.5.990S

[3] J. E. Morley , “Aspects of the Medical History Unique to Older Persons,” JAMA 269 (1993): 677–678. 10.1001/jama.269.5.675 8421372

[4] S. von Haehling , J. E. Morley , and S. D. Anker , “From Muscle Wasting to Sarcopenia and Myopenia: Update 2012,” Journal of Cachexia, Sarcopenia and Muscle 3, no. 4 (2012): 213–217, 10.1007/s13539-012-0089-z PMID: 23160774; PMCID: PMC3505577. 23160774 PMC3505577

[5] W. J. Evans , J. Guralnik , P. Cawthon , et al., “Sarcopenia: No Consensus, No Diagnostic Criteria, and No Approved Indication‐How Did We Get Here?,” Geroscience 46, no. 1 (2024): 183–190, 10.1007/s11357-023-01016-9 Epub 2023 Nov 24. PMID: 37996722; PMCID: PMC10828356. 37996722 PMC10828356

[6] L. M. Baehr , D. W. West , G. Marcotte , et al., “Age‐Related Deficits in Skeletal muscle Recovery Following Disuse Are Associated With Neuromuscular Junction Instability and ER Stress, Not Impaired Protein Synthesis,” Aging (Albany NY) 8, no. 1 (2016): 127–146, 10.18632/aging.100879. PMID: 26826670; PMCID: PMC4761718. 26826670 PMC4761718

[7] B. H. Goodpaster , S. W. Park , T. B. Harris , et al., “The Loss of Skeletal Muscle Strength, Mass, and Quality in Older Adults: The Health, Aging and Body Composition Study,” Journals of Gerontology. Series A, Biological Sciences and Medical Sciences 61, no. 10 (2006): 1059–1064, 10.1093/gerona/61.10.1059 PMID: 17077199. 17077199

[8] P. R. Carvalho do Nascimento , S. Poitras , and M. Bilodeau , “How Do We Define and Measure Sarcopenia? Protocol for a Systematic Review,” Systematic Reviews 7, no. 1 (2018): 51, 10.1186/s13643-018-0712-y PMID: 29587829; PMCID: PMC5870090. 29587829 PMC5870090

[9] J. Zanker , S. Patel , T. Blackwell , et al., “Osteoporotic Fractures in Men (MrOS) Study Group. Walking Speed and Muscle Mass Estimated by the D_3_‐Creatine Dilution Method Are Important Components of Sarcopenia Associated With Incident Mobility Disability in Older Men: A Classification and Regression Tree Analysis,” Journal of the American Medical Directors Association 21, no. 12 (2020): 1997–2002.e1, 10.1016/j.jamda.2020.03.017 Epub 2020 May 4. PMID: 32381425; PMCID: PMC8057698. 32381425 PMC8057698

[10] J. Zanker , T. Blackwell , S. Patel , et al., “Osteoporotic Fractures in Men (MrOS) Study Group. Factor Analysis to Determine Relative Contributions of Strength, Physical Performance, Body Composition and Muscle Mass to Disability and Mobility Disability Outcomes in Older Men,” Experimental Gerontology 161 (2022): 111714, 10.1016/j.exger.2022.111714 Epub 2022 Jan 29. PMID: 35104566; PMCID: PMC8932551. 35104566 PMC8932551

[11] K. Fearon , W. J. Evans , and S. D. Anker , “Myopenia‐A New Universal Term for Muscle Wasting,” Journal of Cachexia, Sarcopenia and Muscle 2, no. 1 (2011): 1–3, 10.1007/s13539-011-0025-7 Epub 2011 Mar 25. PMID: 21475620; PMCID: PMC3063883. 21475620 PMC3063883

[12] H. T. Langer , A. A. Mossakowski , K. Baar , et al., “Commentaries on Viewpoint: Rejuvenation of the Term Sarcopenia,” Journal of Applied Physiology (Bethesda, MD: 1985) 126, no. 1 (2019): 257–262, 10.1152/japplphysiol.00816.2018 PMID: 30694711; PMCID: PMC6442659. 30694711 PMC6442659

[13] W. J. Evans , M. Hellerstein , R. J. Butterfield , et al., “Reductions in Functional Muscle Mass and Ability to Ambulate in Duchenne Muscular Dystrophy From Ages 4 to 24 years,” Journal of Physiology 602, no. 19 (2024): 4929–4939, 10.1113/JP287069 Epub 2024 Aug 31. PMID: 39216089. 39216089

[14] D. C. Hughes , M. A. Wallace , and K. Baar , “Effects of Aging, Exercise, and Disease on Force Transfer in Skeletal Muscle,” American Journal of Physiology. Endocrinology and Metabolism 309, no. 1 (2015): E1–E10, 10.1152/ajpendo.00095.2015 Epub 2015 May 12. PMID: 25968577; PMCID: PMC4490334. 25968577 PMC4490334

[15] S. Kwon , S. Perera , M. Pahor , et al., “What Is a Meaningful Change in Physical Performance? Findings From a Clinical Trial in Older Adults (the LIFE‐P Study),” Journal of Nutrition, Health & Aging 13, no. 6 (2009): 538–544, 10.1007/s12603-009-0104-z PMID: 19536422; PMCID: PMC3100159. PMC3100159 19536422

[16] S. Perera , S. H. Mody , R. C. Woodman , and S. A. Studenski , “Meaningful Change and Responsiveness in Common Physical Performance Measures in Older Adults,” Journal of the American Geriatrics Society 54, no. 5 (2006): 743–749, 10.1111/j.1532-5415.2006.00701.x PMID: 16696738. 16696738

[17] T. G. Travison , S. Basaria , T. W. Storer , et al., “Clinical Meaningfulness of the Changes in Muscle Performance and Physical Function Associated With Testosterone Administration in Older Men With Mobility Limitation,” Journals of Gerontology. Series A, Biological Sciences and Medical Sciences 66, no. 10 (2011): 1090–1099, 10.1093/gerona/glr100 Epub 2011 Jun 22. PMID: 21697501; PMCID: PMC3202898. 21697501 PMC3202898

[18] R. A. Fielding , W. J. Rejeski , S. Blair , et al., “The Lifestyle Interventions and Independence for Elders Study: Design and Methods,” Journals of Gerontology. Series A, Biological Sciences and Medical Sciences 66, no. 11 (2011): 1226–1237, 10.1093/gerona/glr123 Epub 2011 Aug 8. PMID: 21825283; PMCID: PMC3193523. 21825283 PMC3193523

[19] M. Pahor , J. M. Guralnik , W. T. Ambrosius , et al., “Effect of Structured Physical Activity on Prevention of Major Mobility Disability in Older Adults: The LIFE Study Randomized Clinical Trial,” Journal of the American Medical Association 311, no. 23 (2014): 2387–2396, 10.1001/jama.2014.5616 PMID: 24866862; PMCID: PMC4266388. 24866862 PMC4266388

[20] R. Bernabei , F. Landi , R. Calvani , et al., “Multicomponent Intervention to Prevent Mobility Disability in Frail Older Adults: Randomised Controlled Trial (SPRINTT Project),” BMJ 377 (2022): e068788, 10.1136/bmj-2021-068788 PMID: 35545258; PMCID: PMC9092831. 35545258 PMC9092831

[21] L. Servais , D. Eggenspieler , M. Poleur , et al., “First Regulatory Qualification of a Digital Primary Endpoint to Measure Treatment Efficacy in DMD,” Nature Medicine 29 (2023): 2391–2392, 10.1038/s41591-023-02459-5. 37814063

[22] A. S. Dobs , R. V. Boccia , C. C. Croot , et al., “Effects of Enobosarm on Muscle Wasting and Physical Function in Patients With Cancer: A Double‐Blind, Randomised Controlled Phase 2 Trial,” Lancet Oncology 14, no. 4 (2013): 335–345, 10.1016/S1470-2045(13)70055-X. Epub 2013 Mar 14. PMID: 23499390; PMCID: PMC4898053. 23499390 PMC4898053

[23] J. D. Groarke , J. Crawford , S. M. Collins , et al., “Ponsegromab for the Treatment of Cancer Cachexia,” New England Journal of Medicine 391, no. 24 (2024): 2291–2303, 10.1056/NEJMoa2409515 Epub 2024 Sep 14. PMID: 39282907. 39282907

[24] J. S. Temel , A. P. Abernethy , D. C. Currow , et al., “Anamorelin in Patients With Non‐Small‐Cell Lung Cancer and Cachexia (ROMANA 1 and ROMANA 2): Results From Two Randomised, Double‐Blind, Phase 3 Trials,” Lancet Oncology 17, no. 4 (2016): 519–531, 10.1016/S1470-2045(15)00558-6 Epub 2016 Feb 20. PMID: 26906526. 26906526

[25] S. L. Bonnick , C. C. Johnston, Jr. , M. Kleerekoper , et al., “Importance of Precision in Bone Density Measurements,” Journal of Clinical Densitometry 4, no. 2 (2001): 105–110, 10.1385/jcd:4:2:105 PMID: 11477303. 11477303

[26] P. N. Klassen , V. Baracos , S. Ghosh , L. Martin , M. B. Sawyer , and V. C. Mazurak , “Muscle and Adipose Wasting despite Disease Control: Unaddressed Side Effects of Palliative Chemotherapy for Pancreatic Cancer,” Cancers (Basel) 15, no. 17 (2023): 4368, 10.3390/cancers15174368 PMID: 37686641; PMCID: PMC10486774. 37686641 PMC10486774

[27] G. Caminiti , M. Volterrani , F. Iellamo , et al., “Effect of Long‐Acting Testosterone Treatment on Functional Exercise Capacity, Skeletal Muscle Performance, Insulin Resistance, and Baroreflex Sensitivity in Elderly Patients With Chronic Heart Failure a Double‐Blind, Placebo‐Controlled, Randomized Study,” Journal of the American College of Cardiology 54, no. 10 (2009): 919–927, 10.1016/j.jacc.2009.04.078 PMID: 19712802. 19712802

[28] G. W. P. da Fonseca , R. Sato , M. J. de Nazaré Nunes Alves , and S. von Haehling , “Current Advancements in Pharmacotherapy for Cancer Cachexia,” Expert Opinion on Pharmacotherapy 24, no. 5 (2023): 629–639, 10.1080/14656566.2023.2194489 Epub 2023 Mar 30. PMID: 36995115. 36995115

[29] J. Crawford , C. M. Prado , M. A. Johnston , et al., “Study Design and Rationale for the Phase 3 Clinical Development Program of Enobosarm, a Selective Androgen Receptor Modulator, for the Prevention and Treatment of Muscle Wasting in Cancer Patients (POWER Trials),” Current Oncology Reports 18, no. 6 (2016): 37, 10.1007/s11912-016-0522-0 PMID: 27138015; PMCID: PMC4853438. 27138015 PMC4853438

[30] P. Rozentryt , S. von Haehling , M. Lainscak , et al., “The Effects of a High‐Caloric Protein‐Rich Oral Nutritional Supplement in Patients With Chronic Heart Failure and Cachexia on Quality of Life, Body Composition, and Inflammation Markers: A Randomized, Double‐Blind Pilot Study,” Journal of Cachexia, Sarcopenia and Muscle 1, no. 1 (2010): 35–42, 10.1007/s13539-010-0008-0 Epub 2010 Oct 26. PMID: 21475692; PMCID: PMC3060643. 21475692 PMC3060643

[31] R. Aquilani , C. Opasich , A. Gualco , et al., “Adequate Energy‐Protein Intake Is Not Enough to Improve Nutritional and Metabolic Status in Muscle‐Depleted Patients With Chronic Heart Failure,” European Journal of Heart Failure 10, no. 11 (2008): 1127–1135, 10.1016/j.ejheart.2008.09.002 Epub 2008 Oct 4. PMID: 18835539. 18835539

[32] G. Charles‐Edwards , N. Amaral , A. Sleigh , et al., “Effect of Iron Isomaltoside on Skeletal Muscle Energetics in Patients With Chronic Heart Failure and Iron Deficiency,” Circulation 139, no. 21 (2019): 2386–2398, 10.1161/CIRCULATIONAHA.118.038516 PMID: 30776909. 30776909

[33] R. Lafont , M. Serova , B. Didry‐Barca , et al., “20‐Hydroxyecdysone Activates the Protective Arm of the RAAS via the MAS Receptor,” Journal of Molecular Endocrinology 68, no. 2 (2021): 77–87, 10.1530/JME-21-0033 PMID: 34825653. 34825653

[34] W. Dioh , C. Tourette , S. Del Signore , et al., “A Phase 1 Study for Safety and Pharmacokinetics of BIO101 (20‐Hydroxyecdysone) in Healthy Young and Older Adults,” Journal of Cachexia, Sarcopenia and Muscle 14, no. 3 (2023): 1259–1273, 10.1002/jcsm.13195 Epub 2023 Apr 13. PMID: 37057316; PMCID: PMC10235879. 37057316 PMC10235879

[35] A. C. McPherron , A. M. Lawler , and S. J. Lee , “Regulation of Skeletal Muscle Mass in Mice by a New TGF‐Beta Superfamily Member,” Nature 387, no. 6628 (1997): 83–90, 10.1038/387083a0 PMID: 9139826. 9139826

[36] D. Rooks , O. Petricoul , J. Praestgaard , M. Bartlett , D. Laurent , and R. Roubenoff , “Safety and Pharmacokinetics of Bimagrumab in Healthy Older and Obese Adults With Body Composition Changes in the Older Cohort,” Journal of Cachexia, Sarcopenia and Muscle 11, no. 6 (2020): 1525–1534, 10.1002/jcsm.12639 Epub 2020 Dec 2. PMID: 33264516; PMCID: PMC7749589. 33264516 PMC7749589

